# Towards a behavioral profile of degree adverbs in Mainland and Taiwan Mandarin news language

**DOI:** 10.1371/journal.pone.0348273

**Published:** 2026-07-23

**Authors:** Yan Xiao, Qiurong Zhao

**Affiliations:** 1 Qinghai University, Xining, China; 2 University of Science and Technology Beijing, Beijing, China; Education University of Hong Kong, HONG KONG

## Abstract

The major difference between Mainland Mandarin and Taiwan Mandarin in adverb usage lies in degree adverbs. Exploring the similarities and differences in degree adverbs across both sides of the strait not only helps reveal the subtle differences between Chinese dialectal varieties, but also helps broaden the path for computer-assisted compilation of language dictionaries, and enlighten the research method used by language variation studies, comparative language studies and other applied linguistics. This paper compares degree adverbs in Mainland Mandarin and Taiwan Mandarin based on self-built news corpus by using BP (behavior profile) approach and finds that: (1) Degree adverbs with high and medium intensification/mitigation level are more commonly used while degree adverbs with extreme and low intensification/mitigation level are avoided on both sides. In addition, Taiwan uses more extreme degree adverbs than Mainland. (2) The usage of degree adverbs in Mainland and Taiwan Mandarin is significantly related to the semantic prosody, word class, and syntactic function of the words they modify. Specifically, in terms of the semantic prosody of the words they modify, degree adverbs in Mainland modify more positive semantic prosody than those in Taiwan. In terms of the word class of the words they modify, degree adverbs in Mainland mostly modify verbs while degree adverbs in Taiwan corpus mostly modify adjectives. In terms of the syntactic function of the words they modify, degree adverbs in Mainland mostly modify predicates while degree adverbs in Taiwan mostly modify attributives. (3) Cluster diagram analysis shows that the unique degree adverbs on both sides can be substituted with degree adverbs that have similar behavioral profile by each other, which reflects the openness and flexibility of language. This article further points out that the similarities and differences between degree adverbs in Mainland and Taiwan are mainly influenced by news language norms, regional socio-cultural differences, and language change and contact. This paper aims to provide new insights for language users regarding the subtle differences between dialectal varieties in news language and beyond, thereby facilitating more effective communication and the appropriate language use.

## 1. Introduction

Due to geographical distance, social norms and linguistic function, dialectal varieties of the same language are produced and formed diachronically [1]. In this regard, Chinese varieties known as 普通话 (pǔ tōng huà *common language*; hereafter Mainland Mandarin) in Mainland and 国语 (guó yǔ *national language*; hereafter Taiwan Mandarin) in Taiwan are formed and their differences and similarities have attracted widespread interest in linguistic studies, including but not limited to phonology, morphology, vocabulary and grammar [2]. Knowing how the similar words are used in these two regions is not only of great importance to unveil the similarities and differences between these two dialectal varieties but also enlighten the studies on other dialectal varieties of the same language methodologically in the future.

Actually, the differences between these two dialectal varieties are mostly lexical while their grammatical aspects are basically the same [3]. At present, there are many studies investigating into lexical differences between these two dialectal varieties. The early-stage studies mainly focus on description of their vocabulary differences such as tense, pronouns and verb-object construction [4], homographs, address words and loanwords [5], synonymy with different sequence and synonymy with isomerism [6] and the dictionary differences in these two regions [7,8]. With the development of corpus linguistics, many researches start to explore the language differences on a large scale of data in a more objective way. Jiang [9] investigates two grammatical constructions—light verb construction and VO_1_ + O_2_ construction in Mainland Mandarin and Taiwan Mandarin. Zhou & Zhou [10] investigate the vocabulary differences such as different name of the same thing and different pronunciation of the same words between Mainland Mandarin and Taiwan Mandarin. Wang & Zhang [11] compare the similarities and differences in the collocates of the word 改变 gǎi biàn *change* in the subject-predicate relation and the verb-object relation based on the large-scale data in Mainland and Taiwan from The Tagged Chinese Gigaword Corpus.

The researches above provide abundant outcomes and useful insights into the Mainland Mandarin and Taiwan Mandarin. However, few studies investigate the similarities and differences of degree adverbs used in Mainland Mandarin and Taiwan Mandarin. Besides, most of the studies above investigate the literature genre of cross-strait language while the subtle language differences and changes are more instantly reflected in news genre [12], which remains to be explored. Last but not the least, language differences are formed by a combination of multiple factors, and language research should also consider the influence of multiple factors [13], while even though some researches are using corpus-based methods they fail to consider into multifactorial influence on language differences. Considering the three gaps above to be filled, based on multifactorial statistical methods, this study aims to find out the accurate similarities and differences in degree adverbs between Mainland Mandarin and Taiwan Mandarin in the news language by answering the following two questions:

1. What are the similarities and differences of behavioral profile of degree adverbs between Mainland Mandarin and Taiwan Mandarin news language?
2. What are the possible factors that cause the similarities and differences in degree adverbs between Mainland Mandarin and Taiwan Mandarin news language?

## 2. Literature review

### 2.1 Behavioral profile-based studies

Divjak & Gries [14] point out that while there is considerable interest in studying near synonymy, doing so presents substantial challenges from lexical-semantic and synonymy-related perspectives. To solve the problem, BP approach arises based on the assumption of the relationship between the distributional characteristics of a linguistic expression and its semantic and functional properties. Regarding that, both Firth [15] and more explicitly, Harris [16] indicate the importance to explore an expression by investigating into its surroundings. The former [15] says “you shall know a word by the company it keeps”. The later [16] says “if we consider words or morphemes A and B to be more different in meaning than A and C, then we will often find that the distributions of A and B are more different than the distributions of A and C”. More scholars such as Gries & Otani [17] consent those assumptions and indicate that “the distributional characteristics of the use of an item reveal many of its semantic and functional properties and purposes”.

Behavioral profile analysis method is a multifactorial research method [18], which emphasizes the joint influences of various linguistic features such as morphology, syntax, semantics and pragmatics on word choice. It is widely used in near synonymous studies recently with the development of corpus-based methods. Divjak & Gries [19] use the BP approach to study near-synonymous phasal verbs—*begin* and *start* in English and Russian. Gries & Otani [17] investigate size adjectives in English—*big*, *large*, and *great*—and a set of adjectives antonymous to those—*little*, *small*, and *tiny*—plus all their morphological forms attested in the ICE-GB.

BP approach is also used in polysemy studies to differentiate the various usages of a same word in different context. Berez & Gries [20] apply this method to a highly polysemous verb—*to get* in English and reveal how the various senses of the verb relate to one another. Jansegers et al. [21] investigate the intricate polysemy of the Spanish perception verb sentir *feel*, which is analogous to the more extensively studied visual perception verbs ver *see* and mirar *look*, and which also exhibits a wide range of semantic uses across diverse syntactic environments.

In addition to synchronic studies of near-synonymous words and polysemy, the BP approach has also been applied in diachronic research to explore the semantic changes of individual words over time. For example, Jansegers & Gries [22] examine the diachronic evolution of the polysemy of the Spanish verb sentir *feel* by BP approach. Liu [23] adopts a corpus-based behavioral profile approach, combining multifactorial usage-feature analysis with frequency-based quantitative analysis, to investigate the diachronic semasiological variation of the Mandarin Chinese temperature term 热 re *hot*.

Overall, BP approach as a corpus-based multifactorial research approach can be utilized for exploring various linguistic phenomena. The aforementioned studies demonstrate its efficacy in distinguishing expressions with nuanced differences. Applying multifactorial analysis to investigate the textual or even contextual factors that language is subject to is conducive to the development of language research [13,24]. However, existing research primarily employs the BP approach in synchronic or diachronic studies to explore semantic differences within a single language or between different languages. Cross-strait dialectal varieties, which often display more similarities than differences [25], make the BP approach particularly suitable for examining these subtle distinctions. In this regard, BP analysis can contribute to investigate register-specific variation, second-language language and native language, or dialectal varieties of a same language. However, few studies use BP approach to compare the differences between degree adverbs in different genres or dialectal varieties of the same language.

### 2.2 Studies on degree adverbs

Degree adverbs are a category of adverb used to indicate the intensity of a property or state, or the extent of certain actions or behaviors [26]. They typically occur after the subject or after the topic if there is no subject [27]. According to the presence or absence of comparison objects, degree adverbs can be divided into relative degree adverbs and absolute degree adverbs [28]. With the advancement of research and the development of mandarin, some scholars further classify the intensification/mitigation level of both relative degree adverbs and absolute degree adverbs into extreme, high, medium and low [29,30]. Additionally, other scholars expand this classification to include degree adverbs with shifting meanings [31,32]. Degree adverbs are an indispensable part of any language system, as they help express meaning accurately and specifically, and they are widely used across languages and communicative contexts.

The early-stage studies of degree adverbs mainly explore one of their semantical or syntactical aspects. For example, Peters [33] examines the semantic aspect of the degree adverbs, as well as their incidence and distribution in letters from Early modern English. Zhou [34] compares semantic, syntactic and pragmatic aspects between Chinese absolute degree adverbs and relative degree adverbs. Abeillé & Godard [35] explore and assign French degree adverbs two different grammatical functions, adjuncts and complements. Abeillé et al. [36] deal with two types of adverbs that can have a quantificational interpretation: frequency adverbials that quantify over times or events (souvent *often*, parfois *sometimes*, de temps en temps *from time to time*) and degree adverbs, that can have a quantificational effect (beaucoup *a lot*, trop *too much*, complètement *completely*). Doetjes [37] develops an account of the distributional and semantic properties of French degree adverbs beaucoup *a lot* as opposed to frequency adverbs souvent *often*. González-Díaz [38] explores the stylistic stratification of degree adverb *quite* used in different genders. Zhiber & Korotina [39] compare the degree adverbs in spoken corpus and written corpus and find that degree adverbs are used more in spoken corpus than in written corpus.

Apart from the exploration of semantic or syntactic properties of degree adverbs, some scholars focus on how the degree adverbs are shaped across time, such as the grammaticalization or categorization mechanism of degree adverbs. Pertejo [40] investigates the evolution mechanism of degree adverb *absolutely* from a degree adverb to become an independent item used as an emphatic, affirmative response. Visser & Hoeksema [41] trace the grammaticalization process by comparing the degree adverbs in Early Middle Dutch with those in Modern Dutch. Li & Gao [42] describe three ways in which adjectives evolve into degree adverbs. Yang [43] analyzes the categorization phenomenon of the overlapping use of degree adverbs.

Some studies investigate degree adverbs based on behavioral profile analysis approach, Liu & Espino [44] examine the semantic and usage differences among *actually*, *genuinely*, *really*, and *truly*, four near-synonymous adverbs notorious for their complex functional and syntactic usage patterns. Huang & Chen [45] investigate the usage patterns of four near-synonymous degree adverb constructions (i.e., *hěn, tài, mán*, and *chāo*) in the Taiwan Mandarin corpus from TalkBank. However, few studies use this method to explore the nuanced linguistic differences between dialectal varieties of a same language such as Mainland Mandarin and Taiwan Mandarin. Moreover, whether the same patterns occur in other genres, such as news language, remains to be investigated.

Diao [46] points out that the major difference in adverb usage between Mainland Mandarin and Taiwan Mandarin lies in degree adverbs. He further notes that degree adverbs in Taiwan Mandarin modify verbs and nouns more broadly than that in Mainland Mandarin. Zhang [47] points out that the degree adverb 比较 bǐ jiào in Taiwan Mandarin is not restricted from modifying negative structures, which differs from its usage in Mainland Mandarin. However, those early-stage studies are introspective and lack of empirical supports. Fang [48] conducts a statistical analysis of four distinctive degree adverbs (比较 bǐ jiào, 蛮 mán, 好 hǎo, 超 chāo) in spoken Taiwan Mandarin and described their common syntactic structures. Li [49] comparatively analyzes 16 relative and absolute degree adverbs in spoken corpora in Mainland and Taiwan, examining differences and similarities in their frequency and collocational patterns, and further explores their diachronic changes through frequency comparison. Hu [50] compares the frequency with which 比较 bǐ jiào modifies negative structures in Mainland and Taiwan Mandarin, and confirms that the adverb is indeed used more frequently with negative structures in Taiwan than in Mainland. Rui et al. [51] summarize the properties and features of the usages of 老 lǎo in Mainland and 超chāo in Taiwan from the semantic, grammatical and pragmatic perspectives and find that they are highly similar in terms of usages and they both belong to the sub-highest degree adverbs of the absolute degree adverbs with some exceptions.

Overall, research on degree adverbs has made notable progress in both methodological approaches and research scope. However, there are still gaps to be filled. First, many studies focus on only a single linguistic aspect of degree adverbs, whereas a multifactorial analysis is needed to enhance their explanatory power, in line with the contextual coexistence assumption in lexicology. Secondly, the studies on degree adverbs in the dialectal varieties needs further exploration due to its coverage of limited number of degree adverbs. This study aims to cover as much degree adverbs as possible in news corpus which can instantly represent current language usage, with BP approach to investigate the subtle differences between dialectal varieties of the same language.

## 3. Methodology

### 3.1 Composition of corpus

McLaughlin [12] notes that the news genre functions as a site of language contact. The language used in news can immediately reflect the current state of a language. Moreover, it stands out from other genres because it is closely tied to social functions, dissemination mechanisms, and political power. Using news language as a corpus source therefore provides deeper insights into the relationship between language and sociocultural factors. Investigating into news language can not only help us know the quo statue of the language but also a niche material to language variation study [52]. In this regard, news reports are selected as the genre of the corpus in this paper. The corpus consists of two sub-corpora collected from the Mainland newspaper *People’s Daily* and the Taiwan newspaper *China Times. People’s Daily* ranks in top ten of the newspaper in the world for its popularity and reporting scale. *China Times* is the first and foremost newspaper in Taiwan and it has a long broadcasting history and numerous readers. We build news corpus by collecting the news reports released both in 2022 on the website. Specifically, 2022 is selected because it is a recent year that reflects current news language characteristics. No data from other years are included since it is a synchronic study which focuses on a specific static time point. In addition, to compare the language differences in news in general, each topic on these two news websites is collected on the same day to keep the topic balanced and avoid topic bias that may influence the use of degree adverbs. Due to constraints and to maintain a manageable corpus size, the final dataset comprises 329,391 characters and the detailed information is shown in Table 1. We confirm that the collection and analysis of this data fully complied with the terms and conditions of the data sources.

**Table 1:** Composition of news corpus.

District	Source	Time	Size
Mainland	*People’s Daily*	2022	159925
Taiwan	*China Times*	2022	169466
Total	/	/	329391

### 3.2 Degree adverb extraction

The selection of adverbs is initially based on the *Proficiency Level Outline for Chinese Characters* [53]. Even though it lists as much as 31 types of degree adverbs, its scope is still limited to second language vocabulary standards and its representativeness and applicability are relatively restricted. In this regard, based on observation, this study further supplements the list with two high-frequency degree adverbs 真 zhēn *really* and 超 chāo *ultra* to improve the validity of the wordlist. In this way, 33 types of degree adverbs are identified and retrieved for quantitative analysis in the present study.

We find 604 degree adverbs in Mainland and 910 degree adverbs in Taiwan. After filtering out invalid data, such as 维持好 wéi chí hǎo *maintained*, 担任过 dān rèn guò *served*, 非常时期 fēi cháng shí qī *extraordinary period*, 较去年 jiào qù nián *compared with last year*, 346 degree adverbs were obtained in the Mainland corpus and 519 degree adverbs (22 types) were obtained in the Taiwan corpus (22 types), with 16 types shared, and their normalized frequency as occurrences per 100,000 words are further calculated, as shown in Table 2.

**Table 2:** Degree adverbs in Mainland Mandarin and Taiwan Mandarin.

Degree adverb	Mainland Mandarin		Taiwan Mandarin	
	Frequency	Normalized frequency	Frequency	Normalized frequency
更 gèng *further*	105	66	94	55
较 jiào *relatively*	52	33	55	32
很 hěn *very*	42	26	86	51
最 zuì *most*	40	25	86	51
更加 gèng jiā *even more*	34	21	17	10
比较 bǐ jiào *comparatively*	16	10	31	18
非常 fēi cháng *very much*	14	9	36	21
特别 tè bié *especially*	11	7	8	5
十分 shí fēn *highly*	9	6	13	8
极 jí *super*	6	4	11	6
过 guò *excessively*	3	2	5	3
稍 shāo *slightly*	2	1	5	3
太 tài *too*	2	1	32	19
极其 jí qí *extremely*	2	1	0	0
尤其 yóu qí *especially*	2	1	0	0
格外 gé wài *particularly*	1	1	0	0
过于 guò yú *overly*	1	1	2	1
顶 dǐng *extremely*	1	1	0	0
分外 fèn wài *exceptionally*	1	1	0	0
颇 pō *considerably*	1	1	5	3
挺 tǐng *rather*	1	1	2	1
愈 yù *increasingly*	0	0	15	9
稍微 shāo wēi *a little*	0	0	4	2
极度 jí dù *exceedingly*	0	0	1	1
超 chāo *ultra*	0	0	6	4
好 hǎo *quite*	0	0	3	2
真 zhēn *really*	0	0	2	1
Total	346	216	519	30

### 3.3 Degree adverb annotation

Behavioral Profile approach adopts ID tags as linguistic profiles of the expression to be explored. ID tags include morphological, syntactic, semantic, pragmatic and other linguistic features [54]. In order to investigate the multi factors that influence the usage of degree adverbs, we choose three linguistic properties related to the usage of degree adverbs based on previous researches [28,29]. The first feature we annotate is the semantic prosody of the words modified by degree adverbs, namely positive, negative and neutral. The second feature we choose is the word class of the word modified by degree adverbs, which includes verbs, adjectives and nouns. The third feature is syntactic function of the word modified by degree adverbs, which includes predicate, attributive, subject, object, complement, absolute, head, adverbial and predicative.

### 3.4 Statistical methods

In the first part of the research finding, we compare the relativity and intensification/mitigation level of degree adverbs between Mainland Mandarin and Taiwan Mandarin.

In the second part, we examine three linguistic features at the semantic and syntactic levels to investigate the correlation between the usage of degree adverbs and the semantic prosody, word class and syntactic function of the modified word. Through correlation analysis, we can know whether the use of degree adverbs is significantly related to these three factors.

In the third part, we use the multifactorial behavioral analysis method to investigate the similarities between each degree adverb in the corpus. Through hierarchical cluster analysis, we explore the similarity of degree adverbs between Mainland Mandarin and Taiwan Mandarin.

## 4. Findings

### 4.1 Comparation of relativity and intensification/mitigation level of degree adverbs between Mainland Mandarin and Taiwan Mandarin

We calculate the degree adverbs in terms of their token frequency in our corpus based on classification criteria of relative degree adverbs and absolute degree adverbs [28] and extreme, high, medium and low level of intensification/mitigation [29,30], see Table 3.

**Table 3:** Intensification/mitigation level of degree adverbs in Mainland and Taiwan Mandarin.

Degree		Degree adverbs	Mainland Mandarin	Taiwan Mandarin
Relative degree adverbs	extreme	最 zuì *most*	11.56%	16.57%
	high	更 gèng *further,* 更加 gèng jiā *even more*, 格外 gé wài *particularly,* 愈yù *increasingly*	40.46%	24.28%
	medium	较 jiào *relatively,* 比较bǐ jiào *comparatively*	19.65%	16.57%
	low	稍 shāo *slightly,* 稍微shāo wēi *a little*	0.58%	1.73%
Absolute degree adverbs	extreme	极 jí *super*, 太 tài *too*, 极度 jí dù *exceedingly*, 过 guò *excessively*, 过于guò yú *overly*, 极其jí qí *extremely*, 万分wàn fēn *extremely*, 顶dǐng *extremely*, 超chāo *ultra*, 好hǎo *quite*	4.62%	11.56%
	high	非常 fēi cháng *very much*, 十分 shí fēn *highly*, 颇pō *considerably*, 尤其yóu qí *especially*, 真zhēn *really*	7.51%	10.79%
	medium	很 hěn *very*, 挺tǐng *rather*, 特别tè bié *especially*	15.61%	18.50%
	low	有（一）点儿yǒu (yī) diǎnr *a bit*, 有一些yǒu yī xiē *somewhat*	0.00%	0.00%

It can be seen that in terms of token frequency 83.24% of degree adverbs in Mainland Mandarin and 70.14% in Taiwan Mandarin fall into the high and medium categories, while only 16.76% in Mainland Mandarin and 29.86% in Taiwan Mandarin belong to the extreme and low categories. This result is related to the language style of newspapers and periodicals, which focus on disseminating true information as neutrally as possible, which leads to choose high and medium degree adverbs, and less use of extreme and low degree adverbs in newspaper corpus as far as possible. However, in general, degree adverbs of extreme level in Taiwan are more than that of the Mainland, which to a certain extent shows that the language style in Taiwan’s newspapers is less more formal but more radical and freer than that of the Mainland.

In addition, it is worth noting that both the Mainland and Taiwan are more focused on using a high number of relative degree adverbs 更 gèng *further*, 更加 gèng jiā *even more*, 格外 gé wài *particularly*, 愈 yù *increasingly*, see example 1 and 2.

Example 1:

更   立体-的   画面维度   还原   人眼真实

gèng   lìtǐ-ATTR   huàmiàn wéidù   huányuán   rényǎn zhēnshí

more   three-DIM-ATTR   frame dimension   restore   human-eye reality

‘More three-dimensional picture dimensions, restoring human-eye realism.’

(Mainland; *People’s Daily*, 2022-11-30)

Example 2:

全面   毫米波   参考流程   让   RF设计   变得   更快

quánmiàn   háomǐbō   cānkǎo-liúchéng   ràng   RF-shèjì biàn dé   gèng-kuài

comprehensive   millimeter-wave   reference-process   let   RF-design   become   more-fast

‘A comprehensive millimeter-wave reference process makes RF design faster.’

(Taiwan; *China Times*, 2022-12-12)

The degree adverb 更 gèng *further* is one of the high degree adverbs commonly used in the corpus of newspapers and periodicals in both Mainland Mandarin and Taiwan Mandarin, as shown in examples 1 and 2, which indicate the present are often compared with the past to showcase its grand occasion and the positive expectations for the future in news discourse. This is different from what Huang & Chen [45] indicate that *hěn* is the most frequently used degree adverb for intensification. It can be attributed to the language style of news corpus to portrait a better world for people. In addition, in this regard, Mainland has made more prominent use of this kind of degree adverbs in newspapers, accounting for 40.46%.

### 4.2 The correlation between the usage of degree adverbs and their modified words in Mainland Mandarin and Taiwan Mandarin

Before using multifactorial analysis, this section examines the correlation between degree adverbs and three identification tags (semantic prosody, word class, and syntactic function of the modified words) in Mainland and Taiwan using single-factor analysis. As for the data do not follow a normal distribution, non-parametric correlation analysis is conducted.

#### 4.2.1 Semantic prosody.

The semantic prosody of the modified word is both significantly correlated with the usage of degree adverb in Mainland (χ2 (40, N = 346) = 126, p < 0.001, Cramer’s V = 0.43), and Taiwan (χ2 (42, N = 519) = 137, p < 0.001, Cramer’s V = 0.36). Specifically, the degree adverbs in Mainland mostly modify the positive predicate component, accounting for 55%, while distribution of the positive and negative words in the Taiwan are basically the same, 41% and 42%, respectively, see example 3 and 4.

Example 3:

好在   外卖-小哥   很   给力   不到 30 分钟   就   送到

hǎo zài   wàimài-xiǎogē   hěn   gěilì   bù dào 30 fēnzhōng   jiù   sòng-dào

fortunately   delivery-guy   very   capable   NEG reach 30 minute   then   deliver-arrive

‘Fortunately, the delivery guy was really efficient, and it arrived in less than 30 minutes.’

(*People’s Daily*, 2022-11-30)

Example 4:

家属   仅   透露   感觉   他   最近   情绪   很   低潮

jiāshǔ   jǐn   tòulù   gǎnjué   tā   zuìjìn   qíngxù   hěn   dīcháo

family   only   reveal   feel   3SG   recent   mood   very   low.tide

‘Family members only revealed that they feel he has been in a low mood recently.’

(*China Times*, 2022-11-30)

47.6%, 31.0% of degree adverb 很 hěn in Mainland respectively modify the positive and negative predicate component as shown in example 3 while in Taiwan 41.5%, 44.6% respectively modify the positive and negative predicate component as shown in example 4. Cui [55] compares the semantic prosody of the degree adverb 比较 bǐ jiào *comparatively* in the news corpus between Mainland Mandarin and Taiwan Mandarin, and found that it is more commonly used to modify positive meanings in Mainland than in Taiwan, which is as the same as this study.

#### 4.2.2 Word class.

The word class of the modified word is significantly correlated with the usage of degree adverb in Mainland (χ2 (40, N = 346) = 149, p < 0.001, Cramer’s V = 0.46) and Taiwan (χ2 (42, N = 519) = 68, p = 0.007, Cramer’s V = 0.26). In Taiwan Mandarin, degree adverbs have a broader range of modifications to verbs and nouns than Mainland Mandarin [46]. However, it is shown that the proportion of adjectives modified by degree adverbs in Taiwan is larger than that in Mainland, 81% and 63%, respectively, and the degree adverbs in Mainland are more widely used to modify verbs than in Taiwan, 31% and 19%, respectively, and 6% of degree adverbs in Mainland modify nouns, while there are no examples of degree adverbs modifying nouns in Taiwan, see example 5 and 6.

Example 5:

消费者   更   看重   产品   带来-的   幸福感

xiāofèi-zhě   gèng   kànzhòng   chǎnpǐn   dàilái-DE   xìngfú-gǎn

consumer   more   value   product   bring-ATTR   sense-of-happiness

‘Consumers place more value on the happiness that products bring.’

(*People’s Daily*, 2022-11-30)

Example 6:

亚太电信   用户-的   通讯品质   只会   更好

Yàtài-Diànxìn   yònghù-DE   tōngxùn-pǐnzhì   zhǐ huì   gèng hǎo

Asia-Pacific-Telecom   user-ATTR   communication-quality   only-will   more-good

‘The communication quality of Asia-Pacific Telecom users will only get better.’

(*China Times*, 2022-11-23)

According to Table 2, 更 gèng *further* is the most common degree adverb in both Mainland and Taiwan, but in Example 5, it modifies the verb 看重 *value* in Mainland, and in Example 6, it modifies the adjective 好 hǎo *quite* in Taiwan.

The results about the word class modified by degree adverbs in this study are not consistent with the results of previous studies [46].This manifests the dynamic nature of language, just as Diao [25] demonstrates that people take a wrong thought because of outdated studies and points out that many research results are different from the previous ones and we should take a diachronic point of view to this linguistic phenomena in specific historical context.

#### 4.2.3 Syntactic function.

The syntactic function of the modified words is significantly correlated with the usage of degree adverb in Mainland (χ2 (160, N = 346) = 324, p < .001, Cramer’s V = 0.34) and Taiwan (χ2 (168, N = 519) = 460, p < .001, Cramer’s V = 0.33). Overall, the results show that in Mainland, degree adverbs mostly modify predicates, accounting for 37%, while in Taiwan, degree adverbs mostly modify attributives, accounting for 31%. Adjectives are the most common attributives grammatically. As shown in example 6 in 4.2.2, 81% of degree adverbs modify adjectives in Taiwan. The fact that degree adverbs are more often used as attributives in Taiwan can to some extent be attributed to the large proportion of adjectives they modify.

### 4.3 Cluster analysis of degree adverb similarity

Hierarchical clustering is different from other clustering methods in that it is a top-down composition, and the closer the two words are clustered, the higher their similarity they have [56]. Clustering was performed using Ward’s minimum variance linkage method (ward. D2) with squared Euclidean distance to measure dissimilarity between adverbs, as shown below.

**Fig 1:**
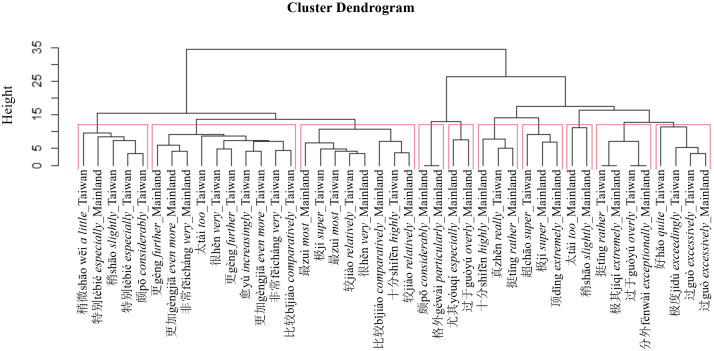
Similarity of degree adverb in Mainland and Taiwan.

Fig 1 shows that clustering tree diagram divides the degree adverbs into ten categories according to their similarity, and the distribution is presented by ten red boxes. In 16 shared degree adverbs in cross-strait corpus, only 7 degree adverbs are grouped into same category, that is 特别 tè bié *especially*, 更加 gèng jiā *even more*, 更 gèng *further*, 非常 fēi cháng *very much*, 最zuì *most*, 较jiào *relatively*, 过guò *excessively*, indicating that the degree adverbs are not very similar in usage.

Degree adverb is one of the most active in terms of undergoing change, and is also a very productive one, since new ‘members’ can be added to it, thus making it an ‘open’ class [40]. The openness also reflect in cross-strait corpus as it have 5 special degree adverbs 格外gé wài *particularly*, 极其jí qí *extremely*, 顶dǐng *extremely*, 分外fèn wài *exceptionally*, 尤其yóu qí *especially* in Mainland and 6 special degree adverbs 愈yù *increasingly*, 稍微shāo wēi *a little*, 极度jí dù *exceedingly*, 超chāo *ultra*, 好hǎo *quite*, 真zhēn *really* in Taiwan. It also can be referred that the usage of degree adverbs in cross-strait newspaper corpus has differentiated with each other and certain degree adverbs are rarely used or not used.

However, in Fig 1, we can see the unique degree adverbs on both sides can be substituted by others with similar behavioral profile in each other’s corpus, which reflects the self-flexibility of language. For example, Mainland-specific 顶dǐng *extremely* is the most similar to Taiwan’s 超chāo *ultra*. At the same time, Taiwan’s unique 真zhēn *really* is most similar to 挺tǐng *rather* in Mainland. Through the clustering analysis of behavior profile of degree adverbs, the degree adverbs with the highest similarity are clustered together, which also provides data support for the dictionary compilation of degree adverbs commonly used in Mainland Mandarin and Taiwan Mandarin.

## 5. Discussion

### 5.1 News language norms

The corpus selected in this study is the corpus of newspapers and periodicals, and the usage of degree adverbs are affected by the language style norms of newspapers and periodicals, showing certain similarities and differences. Newspapers and periodicals are the main media for disseminating current news, introducing people to all aspects of modern economy, politics and culture. The corpus of newspapers and periodicals has the characteristics of being formal, open and oriented to the public, and its language use is more authentic and neutral. The report in broadsheets is reflected differently by using a less biased and involved language than in tabloids [57]. This linguistic style is reflected in the degree adverbs, as both Mainland Mandarin and Taiwan Mandarin use more degree adverbs of high and medium level, and less extreme and low degrees adverbs.

Moreover, degree adverbs such as 分外fèn wài *exceptionally*, 格外gé wài *particularly* is seldom used in both Mainland Mandarin and Taiwan Mandarin, as these two expressions themselves carry a relatively high written-register character, which is not very compatible with news language. The low frequency of some degree adverbs in Mainland Mandarin and Taiwan Mandarin is related to the language style of newspapers and periodicals. In addition, one example of 颇pō *considerably* and no example of 愈yù *increasingly* can be identified in Mainland, while these ancient Chinese degree adverbs are common in Taiwan, which proves that the modern Taiwan Mandarin has inherited and carried forward some words of ancient Chinese in news language, while the news language in Mainland is more colloquial. It can be referred that both Mainland Mandarin and Taiwan Mandarin share a common trend of language characteristics because of the news language style. However, the language style of newspapers and periodicals in both Mainland Mandarin and Taiwan Mandarin still shows certain differences, manifesting its own language style shaped across time and space.

### 5.2 Regional socio-cultural differences

This section discusses the second factor, the socio-cultural norms of newspapers and periodicals in both Mainland Mandarin and Taiwan Mandarin. As Zhou & Zhou [10] put, with lack of information communication during more than 40 years of cross-strait isolation, the social factors are prominent in vocabulary differences. News language has political function through mediating reality and shaping the readers’ ideologies [58,59]. The Mainland corpus in this study comes from the *People’s Daily*, which undertakes “the important responsibilities of understanding social conditions and public opinion, guiding social hot spots, channeling public sentiment, doing a good job in public opinion supervision, disseminating information in various fields at home and abroad in a timely manner, and reporting and commenting on major events in the world” (Introduction to People’s Daily-About Us-People’s Daily Online(people.com.cn)). Influenced by the cultural system of newspapers and periodicals, readers of Mainland newspapers and periodicals are more eager to see positive ideological, cultural and social phenomena. Therefore, the literary wording of Mainland newspapers and periodicals is more conservative, and the degree adverbs mostly modify the positive predicate component. Taiwan corpus in this study is from *China Times*, which is a newspaper created by private companies rather than government agencies, and has repeatedly strained relations with Taiwan authorities over news reports. Taiwan is less affected by the cultural system of newspapers and periodicals, and readers have a more casual attitude towards the things reported by newspapers and periodicals, and have lower positive expectations. Therefore, Taiwan’s newspapers and periodicals are freer in literary speech, and the degree adverbs of positive and negative can be evenly distributed.

### 5.3 Language change and contact

This paper argues that part of the differences between Mainland Mandarin and Taiwan Mandarin in newspapers can be attributed to internal linguistic change. Yang [26] notes that the usage of many degree adverbs in early modern Chinese differs from that in modern Chinese. In early modern Chinese, the most notable feature of degree adverbs is that they can generally modify adjectives relatively freely, whereas their modification of verbs is more restricted. In contrast, in modern Chinese, degree adverbs could modify verbal structures with greater flexibility [26]. The present study finds that the evolution of degree adverbs in Mainland in terms of the word classes they can modify has proceeded significantly faster than that of Taiwan—degree adverbs in Mainland now widely modify verbs. Similarly, Chui [60] observes that the degree adverbs have undergone three developmental stages. Taiwan Mandarin corresponds to the second stage, in which it can largely modify adjectives functioning as attributives, whereas Mainland Mandarin has reached the third stage, in which it can largely modify verbs functioning as predicates.

Moreover, language development is influenced not only by internal changes but also potentially by contact with foreign languages. As Peng & Yan [61] point out, the increase of degree adverbs in the Guangdong region results from the combined effects of foreign linguistic influences, pragmatic needs within Chinese, and the inheritance of the language’s internal developmental patterns. This observation is empirically supported by the findings of the present study. This paper deems that one of the reasons why degree adverbs in Mainland modify mostly verbs while degree adverbs in Taiwan modify mostly adjectives may be that degree adverbs in Mainland are more influenced by translation. As put by Teich [62], in a translation into a given target language (TL), the translation may be oriented more towards the source language (SL), i.e., the SL shines through. The influence of source sentence structure is decreased with increasing translation competence scores [63]. In Taiwan, the promotion of English language proficiency for all people and the use of English everywhere in society have been emphasized, and some high-ranking officials even have states that “English will be developed into a quasi-official language” [64]. Taiwan’s emphasis on improving English proficiency has made second language interference less common. While in Mainland, the English language in education and daily use is not as highlighted as in Taiwan. There is 20 times and 3 times more verbs than adjectives that “genuinely” and “really” respectively modify in COCA (Corpus of contemporary American English) [44]. To a certain extent, this confirms that the degree adverbs modify verb in Mainland more than that in Taiwan is influenced by the English-Chinese translation, resulting in the transfer of language usage features.

## 6. Conclusion

Based on a corpus consisted of news reports in Mainland Mandarin and Taiwan Mandarin, this paper compares their similarities and differences of degree adverbs and finds that, firstly both Mainland Mandarin and Taiwan Mandarin use more high and low intensification/mitigation level of degree adverbs and avoid the use of extreme and low degree adverbs, while Taiwan uses more extreme degree adverbs, indicating that Taiwan’s news language is more colloquial. Secondly the use of degree adverbs in both Mainland Mandarin and Taiwan Mandarin are significantly depended on their semantic prosody, word class and the syntactic function of the words they modify. Additionally, a close comparison of the clustered tree diagrams shows that only a small number of degree adverbs in Mainland Mandarin and Taiwan Mandarin can be grouped together. The unique degree adverbs on each side can often be substituted by other degree adverbs with similar behavioral profiles, reflecting the openness and flexibility of language. Finally, the differences between degree adverbs in these two dialectal varieties can be attributed to news language norms, regional socio-cultural differences, and language change and contact.

In the future, apart from the semantic, syntactic level, the linguistic features of other levels such as discourse features can also be examined in the use of degree adverbs. In terms of corpus to be used, corpora of various genres can be added to further explore the different use of degree adverbs between genres, and diachronic corpus can help explore the diachronic changes in both Mainland Mandarin and Taiwan Mandarin.
