# Supplementary material for: Towards a behavioral profile of degree adverbs in Mainland and Taiwan Mandarin news language
Source: PLoS One. 2026 Jul 23;21(7):e0348273. doi: 10.1371/journal.pone.0348273 (PMC13395354; doi:10.1371/journal.pone.0348273)
Supplement: Attachment — Submitted filename: Response to Reviewers.docx [file pone.0348273.s002.docx]

Thank you very much for your valuable comments, which have greatly contributed to improving this manuscript. We have carefully considered each of your suggestions and made corresponding revisions accordingly.

Reviewer #1: All page and line numbers cited below refer to the PDF version with the “Manuscript Draft” section removed.

① Page 2, lines 3–5
As a study on varieties of Standard Chinese, the manuscript should maintain appropriate political awareness and academic neutrality. The citation of Huang et al. (2014) to characterize the divide between Putonghua and Guoyu is problematic in two respects: first, the claim is not accepted within the academic context of Mainland China; second, the cited source itself cannot be regarded as authoritative. In addition, attributing the lack of cross-strait interaction before 1987 solely to the Jieyanling (martial law) is unnecessary and potentially controversial. This specific causal explanation should be removed, and the issue may be treated in a more general and non-committal manner.
We thank the reviewer for this insightful comment. First, we have ensured political neutrality by revising the relevant discussion and adopting a more generalized description of the language variation. Second, we have replaced the original citation with a more authoritative and widely accepted source. These revisions aim to improve both the academic rigor and the appropriateness of the manuscript.

② Page 3, line 3 (“synonyms such as degree adverbs”)
“Degree adverbs” are defined at the level of word class, whereas “synonyms” belong to the level of semantic relations. These two notions operate on different analytical planes and cannot be used interchangeably or in parallel. The current wording reflects a conceptual confusion and must be revised.

We thank the reviewer for pointing this out. The wording has been revised accordingly: “near synonyms such as degree adverbs” has been replaced with “similarities and differences of degree adverbs” to more accurately reflect the conceptual distinction.

③ Pages 5–7, Section 2.2 “Studies on Degree Adverbs”
The literature review on degree adverbs is highly unrepresentative and omits many foundational scholars and core works in the field, including (but not limited to) Yang Bojun, He Lexi (2001), Tang Xianqing (2004), Yang Rongxiang (2005), Ge Jiacai (2005), Gao Yuhua (2007), Zhang Yisheng, as well as Li & Thompson (1981), Hengeveld (1997), Lyons (1977), Langacker (1987, 1991, 2008), Schachter & Shopen (2007), Cinque (1999, 2004), and Ernst (2002, 2007). Instead, the authors rely heavily on marginal or non-representative studies. This indicates an insufficient grasp of the core theoretical traditions and research trajectory of degree adverb studies.

We appreciate the reviewer’s valuable suggestion. We have consulted the references mentioned and incorporated them into the manuscript. In particular, we have added a review and citations of the works by Yang Bojun, He Lexi (2001), Yang Rongxiang (2005), Ge Jiacai (2005), and Li & Thompson (1981), thereby strengthening the literature review on degree adverbs.

④ Page 8, line 1
The authors must explain why only data from the year 2022 are selected. Specifically, why was 2022 chosen, and why are no data from other years included?
Thank you for your comment. We have clarified in the manuscript the rationale for selecting data from the year 2022.

⑤ Page 8, Section 3.2 “Degree Adverb Extraction,” line 1
The study focuses on degree adverbs in Putonghua and Guoyu, yet it relies on the HSK vocabulary list, which is not the latest version and is primarily designed for teaching Chinese as a second language. Its representativeness and suitability for this research are therefore limited and require explicit justification.
We thank the reviewer for this helpful comment. We have added an explanation in the manuscript clarifying that, while the study is based on the HSK vocabulary list, we supplemented it with manually identified degree adverbs observed in the corpora. This approach ensures that the selection maintains continuity with previous existing list while also being targeted and relevant for the study.

⑥ Page 10, “Degree Adverb Annotation,” first paragraph, lines 3–5
The citation of Knowles et al. (1974) (“Knowles et al. (1974) indicate that functionally degree adverb is the modifier of either an adjective or a verb, like the Manner Adverbial whose distribution it shares, and from which it is to be distinguished merely by lexical feature”) has no clear analytical function in this context. Its relevance to the surrounding discussion is unclear, and the quotation does not contribute substantively to the analysis. The authors should either clarify its purpose or reconsider its inclusion.
We appreciate the reviewer’s suggestion. Upon review, we agree that the citation of Knowles et al. (1974) does not add substantive value in this context, and it has therefore been removed from the manuscript.

⑦ Page 13, “Examples 3 and 4”
These two examples are insufficient to support the claim that degree adverbs in Mainland Mandarin mostly modify positive predicate components (55%), whereas in Taiwan Mandarin the distribution of positive and negative predicates is roughly balanced (41% vs. 42%). More representative examples should be provided to substantiate this conclusion.
We appreciate the reviewer’s comment. The manuscript has been revised to include more representative examples, ensuring that the conclusion about the usage of degree adverbs in mainland and Taiwan is better substantiated.

⑧ Page 13, Section 4.2.2 “Parts of Speech”
First, the correct English term for “词性” is word class, not parts of speech. The manuscript contains multiple instances of literal or machine-translated expressions, which require thorough revision.
Second, the analysis in this subsection is overly simplistic. For example, in “极大的健康影响”, the degree adverb “极大” modifies a verbal phrase rather than a single word. If the modification target is a phrase, word-class-based analysis is no longer applicable, rendering the analysis theoretically invalid.
We thank the reviewer for this insightful comment. The term “Parts of Speech” has been revised to “Word Class” throughout the manuscript. In addition, we have replaced the examples and updated the analysis in this subsection to address the issue raised.

⑨ Page 14, line 14 (“Diao 2000”)
The authors attribute discrepancies between their findings and Diao Yanbin (2000) solely to the time of Diao’s data. However, Diao has published a series of works on Chinese varieties in recent years. The authors should clarify whether these more recent findings align with or diverge from their own results and provide a comparative discussion.
We thank the reviewer for this helpful comment. We have added citations to Diao’s more recent works and used them to clarify that linguistic differences and changes should be considered from a dynamic perspective.

⑩ Page 14, Section “Syntactic Components”
First, the use of the term “Syntactic Components” is incorrect.
Second, in lines 2–3 of this subsection, only the chi-square values and significance levels are reported, rendering the statistical information clearly incomplete. The authors fail to report the degrees of freedom (df), which prevents readers from assessing the structure of the contingency tables and the source of the chi-square statistics. Moreover, given that chi-square tests are highly sensitive to large sample sizes, statistical significance alone is insufficient to reflect the actual strength of the association. Effect size measures (e.g., Cramér’s V or the Phi coefficient) should therefore be reported.
We thank the reviewer for this valuable comment. We have added the degrees of freedom and Cramér’s V to the manuscript to strengthen the scientific rigor of the statistical analysis.

⑪ Page 15, first paragraph below Figure 1
The explanation of the hierarchical clustering method and results in Figure 1 contains serious methodological errors.
First, hierarchical clustering does not represent any temporal process; expressions such as “the earlier/later the two words are clustered” are theoretically untenable. Similarity is indicated by merge height, not by temporal order.
Second, the division into ten clusters is determined by objectively existing semantic categories, and it has nothing to do with time. The conclusions drawn here go beyond what hierarchical clustering can legitimately support.
We appreciate the reviewer’s insightful comment. The original misstatements were due to translation errors from Chinese to English, and the text has been carefully revised to accurately reflect the methodology and results.

⑫ Page 16, last paragraph, line 2
The explanation that “分外 (fèn wài) / 格外 (gé wài) are seldom used because they mainly create a literary atmosphere” is unconvincing. A more reasonable explanation is that these two expressions themselves carry a relatively high written-register character, rather than functioning simply as literary or rhetorical devices.
We appreciate the reviewer’s suggestion. The text has been revised to reflect that the relatively low usage of “分外 (fèn wài)” and “格外 (gé wài)” is due to their inherently high written-register character, rather than their serving merely as literary or rhetorical devices.

⑬ Page 17, lines 7–9 from the bottom
The sentence “It can be seen that Taiwan’s newspapers and periodicals have not established good ties with the local government, and there is a lack of corresponding rules and regulations” has no clear logical connection to the surrounding discussion. Its discourse function is unclear and it should be deleted or substantially revised.
We appreciate the reviewer’s suggestion. The sentence in question has been removed from the manuscript to improve clarity and maintain logical coherence.

⑭ Page 18, lines 1–2
The claim that Mainland degree adverbs modify verbs more often because they are “more influenced by translation” is fundamentally incorrect. Adverbs inherently function as modifiers of VP, and this has nothing to do with translation influence. Pursuing this line of argument would also imply that degree adverbs did not exist in Classical Chinese, which is untenable both theoretically and empirically.
We appreciate the reviewer’s suggestion. This subsection has been revised to clarify that the observed differences in the use of degree adverbs are influenced jointly by language change and language contact, rather than by translation effects alone.

⑮ Page 19, Note 1
All notes should be written in English. The use of Chinese in notes is inappropriate in an English-language article.
We appreciate the reviewer’s suggestion. The note previously written in Chinese has been converted to English to comply with the standards of an English-language manuscript.

⑯ General comments
All examples should follow the Leipzig Glossing Rules, with line-by-line presentation, segmentation, grammatical annotation, and clear indication of corpus sources (e.g., publication name and date).
In addition, Chinese characters are inconsistently presented in simplified and traditional forms. The manuscript should consistently use simplified Chinese throughout.
The manuscript uses Putonghua, Guoyu, Mandarin, and Chinese in a mixed and inconsistent manner. This is a basic terminological problem. The authors must unify their terminology and ensure consistent usage throughout the entire manuscript.

We appreciate the reviewer’s suggestions. The manuscript has been revised so that all examples adhere to the Leipzig Glossing Rules, with line-by-line presentation, segmentation, grammatical annotation, and clear citation of corpus sources. Chinese characters are now presented consistently in simplified form, and terminology has been standardized throughout the manuscript to ensure uniform usage of key terms.

Reviewer #2: This manuscript applies behavioral profile analysis to degree adverbs in cross-strait Chinese news corpora. While the topic is relevant, fundamental methodological weaknesses and analytical superficiality undermine its contributions.

The Abstract claims degree adverbs are "the most different adverbs" between varieties without citation or justification. The Introduction asserts that "subtle differences...are more instantly reflected in news genre" but provides no empirical evidence supporting this genre-specific claim. The rationale for excluding spoken corpora, where degree adverbs naturally vary, remains unexplained, particularly given prior studies (Huang & Chen 2022) demonstrating rich variation in spoken Taiwanese Mandarin.
We appreciate the reviewer’s valuable suggestions. Regarding the first issue, we are not permitted to add a citation in the Abstract. However, to substantiate the claim that degree adverbs exhibit the most variation between varieties, we have retained the relevant citation in Section 2.2 Studies on Degree Adverbs: “Diao[1] points out that the major difference in adverb usage between Mainland Mandarin and Taiwan Mandarin lies in degree adverbs.”

Thanks for the reviewer’s comment, the other two points have been further clarified to better frame the research gap. Although most prior studies focus on literary corpora, news corpora can capture language change more rapidly (McLaughlin 2024, 19), highlighting the need for further investigation. In addition, while the BP method has been applied to spoken corpora (Huang & Chen 2022), it has not yet been applied to news corpora. This strengthens both the rationale and the logical coherence of our study.

Section 2.1 reviews BP applications extensively yet fails to justify why this approach suits cross-strait comparison better than simpler frequency or collocation analyses. The corpus (Section 3.1) comprises only 329,391 characters yielding merely 346 mainland tokens and 519 Taiwan tokens, sample sizes inadequate for robust multifactorial modeling. More critically, the study extracts 33 degree adverbs from a pre-existing list (Proficiency Level Outline 2001) rather than inductively identifying adverbs actually used in the corpus, contradicting BP's inductive ethos. Table 2 reveals severe data sparsity: 11 adverbs have frequencies ≤2, rendering statistical comparison meaningless.
We thank the reviewer for this detailed and constructive comment. We have added a justification in Section 2.1 for using the BP approach in a cross-strait comparison, explaining why it provides advantages to investigate their subtle distinctions. Regarding the corpus size and data sparsity, we acknowledge the limitations and have clarified them in the manuscript. While the study is based on the Proficiency Level Outline (2001) for consistency, we supplemented it by manually verifying and adding degree adverbs actually observed in the corpus, ensures that the initial selection maintains continuity with previous existing list and also align more closely with BP’s inductive approach. These revisions aim to address concerns about sample adequacy, data sparsity, and methodological rigor.

Section 4.1's claim that "83.24% of degree adverbs...fall into high and medium categories" conflates types with tokens, producing misleading percentages. The correlation analyses (Sections 4.2.1–4.2.3) report chi-square results without effect sizes or post-hoc tests, making it impossible to assess whether "significant" differences are substantively meaningful. For instance, stating mainland adverbs "mostly modify positive semantic prosody" (55% vs. Taiwan's 41%) lacks context—are these proportions statistically distinguishable from chance given the small samples? Figure 1's hierarchical clustering groups adverbs with unspecified distance metrics and linkage methods, rendering interpretation arbitrary. The Discussion attributes differences to "translational shining-through effects" (Section 5.3) based solely on speculation about English proficiency levels, without examining actual translation source texts or providing comparative evidence from English corpora beyond a single COCA reference.
We appreciate the reviewer’s insightful comments. The manuscript has been revised to clarify that the reported percentages are based on token frequencies. Effect sizes (Cramér’s V) have been added to the correlation analyses to enhance statistical rigor. Additionally, Section 5.3 has been revised and retitled “Language Change and Contact,” with references to prior studies demonstrating that the observed differences in degree adverb usage between Mainland and Taiwanese Mandarin result from both internal language change and language contact, rather than being attributable solely to translational influence.

The study's contributions remain unclear: findings largely confirm obvious patterns (formal news avoids extreme language) while ignoring whether observed differences exceed noise thresholds in small samples.
