# Supplementary material for: Towards a behavioral profile of degree adverbs in Mainland and Taiwan Mandarin news language
Source: PLoS One. 2026 Jul 23;21(7):e0348273. doi: 10.1371/journal.pone.0348273 (PMC13395354; doi:10.1371/journal.pone.0348273)
Supplement: Attachment — Submitted filename: Response to Reviewer.docx [file pone.0348273.s003.docx]

Thank you for your careful and professional feedback. With these revisions, the manuscript has been significantly improved.

The abstract currently opens with a citation. In general, an abstract should be self-contained and should not include references.
Thank you for your comment. The citation in the abstract has been removed.

The statement “Degree adverbs are the most different adverbs between Mainland Mandarin and Taiwan Mandarin” is presented as an established conclusion, but the source cited for this claim does not seem to justify such a strong formulation. Later in the literature review, the manuscript presents a milder formulation, namely that the major difference in adverb usage lies in degree adverbs. These two claims are not equivalent. The authors should therefore verify the original source carefully, avoid overstating previous scholarship, and revise the wording accordingly.
Thank you for this valuable comment. We have revised the relevant statements accordingly to avoid overstatement and ensure consistency throughout the manuscript.

The discussion of cross-strait differences in degree adverbs relies heavily on relatively old references, while more recent scholarship on Global Chinese, regional variation in Chinese, and corpus-based comparative studies appears to be largely absent. Given the development of this field in recent years, the manuscript should engage more fully with recent research rather than depending mainly on older studies to frame the research problem.

Thank you for your constructive suggestion. We have supplemented the manuscript with relevant recent literature, thereby enhancing its contemporary relevance and academic value.

One of the central problems of the paper is that it mixes lexical categories and syntactic functions. In the abstract, the authors state that the use of degree adverbs is related to “semantic prosody, word class, and part of speech,” and then compare verbs and adjectives on the one hand, and predicates and attributives on the other. However, verbs and adjectives are lexical categories, whereas predicate and attributive are syntactic functions. These are different analytical dimensions and should not be treated as if they were parallel categories. The same problem reappears in the methodology section. The analytical framework therefore needs to be clarified and kept consistent throughout the paper.
Thank you for your insightful comment. We have revised and supplemented the relevant descriptions to make the analytical framework clearer and ensure consistency throughout the manuscript.

In Section 3.3, the second annotated feature is described as “word class,” with categories such as verbs, adjectives, and nouns. However, the third feature is labeled as “part of speech,” even though the categories listed under it, such as predicate, attribute, subject, object, complement, head, and adverbial, are not parts of speech but syntactic functions or syntactic roles. The terminology is therefore incorrect and should be revised accordingly. In addition, the category list itself contains duplication and apparent editing artifacts, which further weakens confidence in the rigor of the annotation design. The authors should redefine each annotation variable clearly and consistently, using theoretically accurate terms.
Thank you for your professional and meticulous comment. We have revised the term "part of speech" to "syntactic function", removed the duplications caused by editing errors, and redefined each annotation variable clearly and consistently with theoretically accurate terminology.

The statements “Degree adverbs in mainland mostly modify verbs while degree adverbs in Taiwan corpus mostly modify adjectives” and “Degree adverbs in mainland mostly modify predicates while degree adverbs in Taiwan mostly modify attributives” are presented side by side, although the first contrast is based on lexical category and the second on syntactic function. Since these two dimensions are not directly comparable, the current phrasing is potentially misleading. The authors should either separate the two analytical dimensions clearly or reformulate the findings using one consistent basis of comparison.
Thank you for your careful comment. We have added further explanations to clarify the two distinct analytical dimensions and ensure the presentation of findings is clear and non-misleading.

In the submission form, the authors indicate that all data are fully available without restriction. However, the statement provided says that “All required files will be provided from our database on request after acceptance.” This does not seem compatible with the journal’s own requirement, which explicitly states that “data available on request” is not sufficient. This is a serious issue that should be corrected.

Thank you for your comment. We have made corresponding revisions and adjusted the statement to "All required files will be provided from our database" to comply with the journal's standards.

There are many awkward or non-idiomatic expressions throughout the paper, such as “investigate into,” “more used,” “in both sides,” “linguistical function,” and “enlighten the language users.” Phrases such as “enlighten the language users” and “further promoting mutual understanding in communication and the language use in general” are too broad and promotional for an academic abstract. The contribution of the study should be stated in more precise scholarly terms. The manuscript would benefit from careful revision by a proficient academic English user.

Thank you for your detailed feedback. We have revised all awkward and non-idiomatic expressions across the manuscript, and refined the presentation of the study’s contributions using more precise scholarly language to meet academic English writing standards.
