# Supplementary material for: Towards a behavioral profile of degree adverbs in Mainland and Taiwan Mandarin news language
Source: PLoS One. 2026 Jul 23;21(7):e0348273. doi: 10.1371/journal.pone.0348273 (PMC13395354; doi:10.1371/journal.pone.0348273)
Supplement: Peer review history

# Peer review: Decision Letter 0

**DOI:** 10.1371/journal.pone.0348273.r001

**Authors:** Jie Wang

3 Feb 2026

PONE-D-26-00164

Towards a Behavioral Profile of Degree Adverbs in Mainland and Taiwan Mandarin News Language

PLOS One

Dear Dr. Zhao,

Thank you for submitting your manuscript to PLOS ONE. After careful consideration, we feel that it has merit but does not fully meet PLOS ONE’s publication criteria as it currently stands. Therefore, we invite you to submit a revised version of the manuscript that addresses the points raised during the review process.

Please submit your revised manuscript by Mar 20 2026 11:59PM. If you will need more time than this to complete your revisions, please reply to this message or contact the journal office at plosone@plos.org. When you're ready to submit your revision, log on to https://www.editorialmanager.com/pone/ and select the 'Submissions Needing Revision' folder to locate your manuscript file.

Please include the following items when submitting your revised manuscript:

- A letter that responds to each point raised by the academic editor and reviewer(s). You should upload this letter as a separate file labeled 'Response to Reviewers'.
- A marked-up copy of your manuscript that highlights changes made to the original version. You should upload this as a separate file labeled 'Revised Manuscript with Track Changes'.
- An unmarked version of your revised paper without tracked changes. You should upload this as a separate file labeled 'Manuscript'.

If you would like to make changes to your financial disclosure, please include your updated statement in your cover letter. Guidelines for resubmitting your figure files are available below the reviewer comments at the end of this letter.

If applicable, we recommend that you deposit your laboratory protocols in protocols.io to enhance the reproducibility of your results. Protocols.io assigns your protocol its own identifier (DOI) so that it can be cited independently in the future. For instructions see: https://journals.plos.org/plosone/s/submission-guidelines#loc-laboratory-protocols. Additionally, PLOS ONE offers an option for publishing peer-reviewed Lab Protocol articles, which describe protocols hosted on protocols.io. Read more information on sharing protocols at https://plos.org/protocols?utm_medium=editorial-email&utm_source=authorletters&utm_campaign=protocols.

We look forward to receiving your revised manuscript.

Kind regards,

Jie Wang, Ph.D.

Academic Editor

PLOS One

Journal Requirements:

When submitting your revision, we need you to address these additional requirements.

1. Please ensure that your manuscript meets PLOS ONE's style requirements, including those for file naming. The PLOS ONE style templates can be found at

https://journals.plos.org/plosone/s/file?id=wjVg/PLOSOne_formatting_sample_main_body.pdf and

https://journals.plos.org/plosone/s/file?id=ba62/PLOSOne_formatting_sample_title_authors_affiliations.pdf

2. In your Methods section, please include additional information about your dataset and ensure that you have included a statement specifying whether the collection and analysis method complied with the terms and conditions for the source of the data.

3. Please update your submission to use the PLOS LaTeX template. The template and more information on our requirements for LaTeX submissions can be found at http://journals.plos.org/plosone/s/latex.

4. We note that your Data Availability Statement is currently as follows: “All relevant data are within the manuscript and its Supporting Information files.”

Please confirm at this time whether or not your submission contains all raw data required to replicate the results of your study. Authors must share the “minimal data set” for their submission. PLOS defines the minimal data set to consist of the data required to replicate all study findings reported in the article, as well as related metadata and methods (https://journals.plos.org/plosone/s/data-availability#loc-minimal-data-set-definition).

For example, authors should submit the following data:

- The values behind the means, standard deviations and other measures reported;

- The values used to build graphs;

- The points extracted from images for analysis.

Authors do not need to submit their entire data set if only a portion of the data was used in the reported study.

If your submission does not contain these data, please either upload them as Supporting Information files or deposit them to a stable, public repository and provide us with the relevant URLs, DOIs, or accession numbers. For a list of recommended repositories, please see https://journals.plos.org/plosone/s/recommended-repositories.

If there are ethical or legal restrictions on sharing a de-identified data set, please explain them in detail (e.g., data contain potentially sensitive information, data are owned by a third-party organization, etc.) and who has imposed them (e.g., an ethics committee). Please also provide contact information for a data access committee, ethics committee, or other institutional body to which data requests may be sent. If data are owned by a third party, please indicate how others may request data access.

5. PLOS requires an ORCID iD for the corresponding author in Editorial Manager on papers submitted after December 6th, 2016. Please ensure that you have an ORCID iD and that it is validated in Editorial Manager. To do this, go to ‘Update my Information’ (in the upper left-hand corner of the main menu), and click on the Fetch/Validate link next to the ORCID field. This will take you to the ORCID site and allow you to create a new iD or authenticate a pre-existing iD in Editorial Manager.

6. Please amend your authorship list in your manuscript file to include author Qiurong Zhao and Yan Xiao.

7. If the reviewer comments include a recommendation to cite specific previously published works, please review and evaluate these publications to determine whether they are relevant and should be cited. There is no requirement to cite these works unless the editor has indicated otherwise.

Additional Editor Comments:

The authors need to address the comments raised by the two reviewers.

[Note: HTML markup is below. Please do not edit.]

Reviewers' comments:

Reviewer's Responses to Questions

**Comments to the Author**

1. Is the manuscript technically sound, and do the data support the conclusions?

The manuscript must describe a technically sound piece of scientific research with data that supports the conclusions. Experiments must have been conducted rigorously, with appropriate controls, replication, and sample sizes. The conclusions must be drawn appropriately based on the data presented.

Reviewer #1: Partly

Reviewer #2: Partly

**********

2. Has the statistical analysis been performed appropriately and rigorously?

Reviewer #1: Yes

Reviewer #2: Yes

**********

3. Have the authors made all data underlying the findings in their manuscript fully available?

The PLOS Data policy requires authors to make all data underlying the findings described in their manuscript fully available without restriction, with rare exception (please refer to the Data Availability Statement in the manuscript PDF file). The data should be provided as part of the manuscript or its supporting information, or deposited to a public repository. For example, in addition to summary statistics, the data points behind means, medians and variance measures should be available. If there are restrictions on publicly sharing data—e.g. participant privacy or use of data from a third party—those must be specified.

Reviewer #1: Yes

Reviewer #2: Yes

**********

4. Is the manuscript presented in an intelligible fashion and written in standard English?

PLOS ONE does not copyedit accepted manuscripts, so the language in submitted articles must be clear, correct, and unambiguous. Any typographical or grammatical errors should be corrected at revision, so please note any specific errors here.

Reviewer #1: Yes

Reviewer #2: Yes

**********

5. Review Comments to the Author

Please use the space provided to explain your answers to the questions above. You may also include additional comments for the author, including concerns about dual publication, research ethics, or publication ethics. (Please upload your review as an attachment if it exceeds 20,000 characters)

Reviewer #1: All page and line numbers cited below refer to the PDF version with the “Manuscript Draft” section removed.

① Page 2, lines 3–5

As a study on varieties of Standard Chinese, the manuscript should maintain appropriate political awareness and academic neutrality. The citation of Huang et al. (2014) to characterize the divide between Putonghua and Guoyu is problematic in two respects: first, the claim is not accepted within the academic context of Mainland China; second, the cited source itself cannot be regarded as authoritative. In addition, attributing the lack of cross-strait interaction before 1987 solely to the Jieyanling (martial law) is unnecessary and potentially controversial. This specific causal explanation should be removed, and the issue may be treated in a more general and non-committal manner.

② Page 3, line 3 (“synonyms such as degree adverbs”)

“Degree adverbs” are defined at the level of word class, whereas “synonyms” belong to the level of semantic relations. These two notions operate on different analytical planes and cannot be used interchangeably or in parallel. The current wording reflects a conceptual confusion and must be revised.

③ Pages 5–7, Section 2.2 “Studies on Degree Adverbs”

The literature review on degree adverbs is highly unrepresentative and omits many foundational scholars and core works in the field, including (but not limited to) Yang Bojun, He Lexi (2001), Tang Xianqing (2004), Yang Rongxiang (2005), Ge Jiacai (2005), Gao Yuhua (2007), Zhang Yisheng, as well as Li & Thompson (1981), Hengeveld (1997), Lyons (1977), Langacker (1987, 1991, 2008), Schachter & Shopen (2007), Cinque (1999, 2004), and Ernst (2002, 2007). Instead, the authors rely heavily on marginal or non-representative studies. This indicates an insufficient grasp of the core theoretical traditions and research trajectory of degree adverb studies.

④ Page 8, line 1

The authors must explain why only data from the year 2022 are selected. Specifically, why was 2022 chosen, and why are no data from other years included?

⑤ Page 8, Section 3.2 “Degree Adverb Extraction,” line 1

The study focuses on degree adverbs in Putonghua and Guoyu, yet it relies on the HSK vocabulary list, which is not the latest version and is primarily designed for teaching Chinese as a second language. Its representativeness and suitability for this research are therefore limited and require explicit justification.

⑥ Page 10, “Degree Adverb Annotation,” first paragraph, lines 3–5

The citation of Knowles et al. (1974) (“Knowles et al. (1974) indicate that functionally degree adverb is the modifier of either an adjective or a verb, like the Manner Adverbial whose distribution it shares, and from which it is to be distinguished merely by lexical feature”) has no clear analytical function in this context. Its relevance to the surrounding discussion is unclear, and the quotation does not contribute substantively to the analysis. The authors should either clarify its purpose or reconsider its inclusion.

⑦ Page 13, “Examples 3 and 4”

These two examples are insufficient to support the claim that degree adverbs in Mainland Mandarin mostly modify positive predicate components (55%), whereas in Taiwan Mandarin the distribution of positive and negative predicates is roughly balanced (41% vs. 42%). More representative examples should be provided to substantiate this conclusion.

⑧ Page 13, Section 4.2.2 “Parts of Speech”

First, the correct English term for “词性” is word class, not parts of speech. The manuscript contains multiple instances of literal or machine-translated expressions, which require thorough revision.

Second, the analysis in this subsection is overly simplistic. For example, in “极大的健康影响”, the degree adverb “极大” modifies a verbal phrase rather than a single word. If the modification target is a phrase, word-class-based analysis is no longer applicable, rendering the analysis theoretically invalid.

Page 14, line 14 (“Diao 2000”)

The authors attribute discrepancies between their findings and Diao Yanbin (2000) solely to the time of Diao’s data. However, Diao has published a series of works on Chinese varieties in recent years. The authors should clarify whether these more recent findings align with or diverge from their own results and provide a comparative discussion.

⑩ Page 14, Section “Syntactic Components”

First, the use of the term “Syntactic Components” is incorrect.

Second, in lines 2–3 of this subsection, only the chi-square values and significance levels are reported, rendering the statistical information clearly incomplete. The authors fail to report the degrees of freedom (df), which prevents readers from assessing the structure of the contingency tables and the source of the chi-square statistics. Moreover, given that chi-square tests are highly sensitive to large sample sizes, statistical significance alone is insufficient to reflect the actual strength of the association. Effect size measures (e.g., Cramér’s V or the Phi coefficient) should therefore be reported.

⑪ Page 15, first paragraph below Figure 1

The explanation of the hierarchical clustering method and results in Figure 1 contains serious methodological errors.

First, hierarchical clustering does not represent any temporal process; expressions such as “the earlier/later the two words are clustered” are theoretically untenable. Similarity is indicated by merge height, not by temporal order.

Second, the division into ten clusters is determined by objectively existing semantic categories, and it has nothing to do with time. The conclusions drawn here go beyond what hierarchical clustering can legitimately support.

⑫ Page 16, last paragraph, line 2

The explanation that “分外 (fèn wài) / 格外 (gé wài) are seldom used because they mainly create a literary atmosphere” is unconvincing. A more reasonable explanation is that these two expressions themselves carry a relatively high written-register character, rather than functioning simply as literary or rhetorical devices.

⑬ Page 17, lines 7–9 from the bottom

The sentence “It can be seen that Taiwan’s newspapers and periodicals have not established good ties with the local government, and there is a lack of corresponding rules and regulations” has no clear logical connection to the surrounding discussion. Its discourse function is unclear and it should be deleted or substantially revised.

⑭ Page 18, lines 1–2

The claim that Mainland degree adverbs modify verbs more often because they are “more influenced by translation” is fundamentally incorrect. Adverbs inherently function as modifiers of VP, and this has nothing to do with translation influence. Pursuing this line of argument would also imply that degree adverbs did not exist in Classical Chinese, which is untenable both theoretically and empirically.

⑮ Page 19, Note 1

All notes should be written in English. The use of Chinese in notes is inappropriate in an English-language article.

⑯ General comments

All examples should follow the Leipzig Glossing Rules, with line-by-line presentation, segmentation, grammatical annotation, and clear indication of corpus sources (e.g., publication name and date).

In addition, Chinese characters are inconsistently presented in simplified and traditional forms. The manuscript should consistently use simplified Chinese throughout.

The manuscript uses Putonghua, Guoyu, Mandarin, and Chinese in a mixed and inconsistent manner. This is a basic terminological problem. The authors must unify their terminology and ensure consistent usage throughout the entire manuscript.

Reviewer #2: This manuscript applies behavioral profile analysis to degree adverbs in cross-strait Chinese news corpora. While the topic is relevant, fundamental methodological weaknesses and analytical superficiality undermine its contributions.

The Abstract claims degree adverbs are "the most different adverbs" between varieties without citation or justification. The Introduction asserts that "subtle differences...are more instantly reflected in news genre" but provides no empirical evidence supporting this genre-specific claim. The rationale for excluding spoken corpora, where degree adverbs naturally vary, remains unexplained, particularly given prior studies (Huang & Chen 2022) demonstrating rich variation in spoken Taiwanese Mandarin.

Section 2.1 reviews BP applications extensively yet fails to justify why this approach suits cross-strait comparison better than simpler frequency or collocation analyses. The corpus (Section 3.1) comprises only 329,391 characters yielding merely 346 mainland tokens and 519 Taiwan tokens, sample sizes inadequate for robust multifactorial modeling. More critically, the study extracts 33 degree adverbs from a pre-existing list (Proficiency Level Outline 2001) rather than inductively identifying adverbs actually used in the corpus, contradicting BP's inductive ethos. Table 2 reveals severe data sparsity: 11 adverbs have frequencies ≤2, rendering statistical comparison meaningless.

Section 4.1's claim that "83.24% of degree adverbs...fall into high and medium categories" conflates types with tokens, producing misleading percentages. The correlation analyses (Sections 4.2.1–4.2.3) report chi-square results without effect sizes or post-hoc tests, making it impossible to assess whether "significant" differences are substantively meaningful. For instance, stating mainland adverbs "mostly modify positive semantic prosody" (55% vs. Taiwan's 41%) lacks context—are these proportions statistically distinguishable from chance given the small samples? Figure 1's hierarchical clustering groups adverbs with unspecified distance metrics and linkage methods, rendering interpretation arbitrary. The Discussion attributes differences to "translational shining-through effects" (Section 5.3) based solely on speculation about English proficiency levels, without examining actual translation source texts or providing comparative evidence from English corpora beyond a single COCA reference.

The study's contributions remain unclear: findings largely confirm obvious patterns (formal news avoids extreme language) while ignoring whether observed differences exceed noise thresholds in small samples.

**********

6. PLOS authors have the option to publish the peer review history of their article (what does this mean?). If published, this will include your full peer review and any attached files.

If you choose “no”, your identity will remain anonymous but your review may still be made public.

**Do you want your identity to be public for this peer review?**  For information about this choice, including consent withdrawal, please see our Privacy Policy.

Reviewer #1: No

Reviewer #2: No

**********

[NOTE: If reviewer comments were submitted as an attachment file, they will be attached to this email and accessible via the submission site. Please log into your account, locate the manuscript record, and check for the action link "View Attachments". If this link does not appear, there are no attachment files.]

To ensure your figures meet our technical requirements, please review our figure guidelines: https://journals.plos.org/plosone/s/figures

You may also use PLOS’s free figure tool, NAAS, to help you prepare publication quality figures: https://journals.plos.org/plosone/s/figures#loc-tools-for-figure-preparation.

NAAS will assess whether your figures meet our technical requirements by comparing each figure against our figure specifications.

# Author comment: Author response to Decision Letter 1

**DOI:** 10.1371/journal.pone.0348273.r002

30 Mar 2026

Thank you for all you have done.

Attachment Submitted filename: Response to Reviewers.docx

# Peer review: Decision Letter 1

**DOI:** 10.1371/journal.pone.0348273.r003

**Authors:** Jie Wang

6 Apr 2026

PONE-D-26-00164R1

Towards a Behavioral Profile of Degree Adverbs in Mainland and Taiwan Mandarin News Language

PLOS One

Dear Dr. Zhao,

Thank you for submitting your manuscript to PLOS ONE. After careful consideration, we feel that it has merit but does not fully meet PLOS ONE’s publication criteria as it currently stands. Therefore, we invite you to submit a revised version of the manuscript that addresses the points raised during the review process.

Please submit your revised manuscript by May 21 2026 11:59PM. If you will need more time than this to complete your revisions, please reply to this message or contact the journal office at plosone@plos.org. When you're ready to submit your revision, log on to https://www.editorialmanager.com/pone/ and select the 'Submissions Needing Revision' folder to locate your manuscript file.

Please include the following items when submitting your revised manuscript:

- A letter that responds to each point raised by the academic editor and reviewer(s). You should upload this letter as a separate file labeled 'Response to Reviewers'.
- A marked-up copy of your manuscript that highlights changes made to the original version. You should upload this as a separate file labeled 'Revised Manuscript with Track Changes'.
- An unmarked version of your revised paper without tracked changes. You should upload this as a separate file labeled 'Manuscript'.

If you would like to make changes to your financial disclosure, please include your updated statement in your cover letter. Guidelines for resubmitting your figure files are available below the reviewer comments at the end of this letter.

If applicable, we recommend that you deposit your laboratory protocols in protocols.io to enhance the reproducibility of your results. Protocols.io assigns your protocol its own identifier (DOI) so that it can be cited independently in the future. For instructions see: https://journals.plos.org/plosone/s/submission-guidelines#loc-laboratory-protocols. Additionally, PLOS ONE offers an option for publishing peer-reviewed Lab Protocol articles, which describe protocols hosted on protocols.io. Read more information on sharing protocols at https://plos.org/protocols?utm_medium=editorial-email&utm_source=authorletters&utm_campaign=protocols.

As the corresponding author, your ORCID iD is verified in the submission system and will appear in the published article. PLOS supports the use of ORCID, and we encourage all coauthors to register for an ORCID iD and use it as well. Please encourage your coauthors to verify their ORCID iD within the submission system before final acceptance, as unverified ORCID iDs will not appear in the published article. *Only*  the individual author can complete the verification step; PLOS staff *cannot*  verify ORCID iDs on behalf of authors.

We look forward to receiving your revised manuscript.

Kind regards,

Jie Wang, Ph.D.

Academic Editor

PLOS One

Journal Requirements:

If the reviewer comments include a recommendation to cite specific previously published works, please review and evaluate these publications to determine whether they are relevant and should be cited. There is no requirement to cite these works unless the editor has indicated otherwise.

Please review your reference list to ensure that it is complete and correct. If you have cited papers that have been retracted, please include the rationale for doing so in the manuscript text, or remove these references and replace them with relevant current references. Any changes to the reference list should be mentioned in the rebuttal letter that accompanies your revised manuscript. If you need to cite a retracted article, indicate the article’s retracted status in the References list and also include a citation and full reference for the retraction notice.

Additional Editor Comments:

The authors need to address the comments of Reviewer 1.

[Note: HTML markup is below. Please do not edit.]

Reviewers' comments:

Reviewer's Responses to Questions

**Comments to the Author**

1. If the authors have adequately addressed your comments raised in a previous round of review and you feel that this manuscript is now acceptable for publication, you may indicate that here to bypass the “Comments to the Author” section, enter your conflict of interest statement in the “Confidential to Editor” section, and submit your "Accept" recommendation.

Reviewer #1: (No Response)

**********

2. Is the manuscript technically sound, and do the data support the conclusions?

The manuscript must describe a technically sound piece of scientific research with data that supports the conclusions. Experiments must have been conducted rigorously, with appropriate controls, replication, and sample sizes. The conclusions must be drawn appropriately based on the data presented.

Reviewer #1: Partly

**********

3. Has the statistical analysis been performed appropriately and rigorously?

Reviewer #1: Yes

**********

4. Have the authors made all data underlying the findings in their manuscript fully available?

The PLOS Data policy requires authors to make all data underlying the findings described in their manuscript fully available without restriction, with rare exception (please refer to the Data Availability Statement in the manuscript PDF file). The data should be provided as part of the manuscript or its supporting information, or deposited to a public repository. For example, in addition to summary statistics, the data points behind means, medians and variance measures should be available. If there are restrictions on publicly sharing data—e.g. participant privacy or use of data from a third party—those must be specified.

Reviewer #1: Yes

**********

5. Is the manuscript presented in an intelligible fashion and written in standard English?

PLOS ONE does not copyedit accepted manuscripts, so the language in submitted articles must be clear, correct, and unambiguous. Any typographical or grammatical errors should be corrected at revision, so please note any specific errors here.

Reviewer #1: Yes

**********

6. Review Comments to the Author

Please use the space provided to explain your answers to the questions above. You may also include additional comments for the author, including concerns about dual publication, research ethics, or publication ethics. (Please upload your review as an attachment if it exceeds 20,000 characters)

Reviewer #1: The authors have clearly made a serious and conscientious effort to revise the manuscript in response to the first-round review comments, and most of the issues raised previously have been addressed to varying degrees. This suggests that substantial work has gone into improving the paper. However, despite these efforts, the current version still contains a number of important problems in its conceptual framework, literature support, annotation design, compliance with journal requirements, and academic writing. In its present form, the manuscript still requires minor revision before it can be considered further.

The abstract currently opens with a citation. In general, an abstract should be self-contained and should not include references.

The statement “Degree adverbs are the most different adverbs between Mainland Mandarin and Taiwan Mandarin” is presented as an established conclusion, but the source cited for this claim does not seem to justify such a strong formulation. Later in the literature review, the manuscript presents a milder formulation, namely that the major difference in adverb usage lies in degree adverbs. These two claims are not equivalent. The authors should therefore verify the original source carefully, avoid overstating previous scholarship, and revise the wording accordingly.

The discussion of cross-strait differences in degree adverbs relies heavily on relatively old references, while more recent scholarship on Global Chinese, regional variation in Chinese, and corpus-based comparative studies appears to be largely absent. Given the development of this field in recent years, the manuscript should engage more fully with recent research rather than depending mainly on older studies to frame the research problem.

One of the central problems of the paper is that it mixes lexical categories and syntactic functions. In the abstract, the authors state that the use of degree adverbs is related to “semantic prosody, word class, and part of speech,” and then compare verbs and adjectives on the one hand, and predicates and attributives on the other. However, verbs and adjectives are lexical categories, whereas predicate and attributive are syntactic functions. These are different analytical dimensions and should not be treated as if they were parallel categories. The same problem reappears in the methodology section. The analytical framework therefore needs to be clarified and kept consistent throughout the paper.

In Section 3.3, the second annotated feature is described as “word class,” with categories such as verbs, adjectives, and nouns. However, the third feature is labeled as “part of speech,” even though the categories listed under it, such as predicate, attribute, subject, object, complement, head, and adverbial, are not parts of speech but syntactic functions or syntactic roles. The terminology is therefore incorrect and should be revised accordingly. In addition, the category list itself contains duplication and apparent editing artifacts, which further weakens confidence in the rigor of the annotation design. The authors should redefine each annotation variable clearly and consistently, using theoretically accurate terms.

The statements “Degree adverbs in mainland mostly modify verbs while degree adverbs in Taiwan corpus mostly modify adjectives” and “Degree adverbs in mainland mostly modify predicates while degree adverbs in Taiwan mostly modify attributives” are presented side by side, although the first contrast is based on lexical category and the second on syntactic function. Since these two dimensions are not directly comparable, the current phrasing is potentially misleading. The authors should either separate the two analytical dimensions clearly or reformulate the findings using one consistent basis of comparison.

In the submission form, the authors indicate that all data are fully available without restriction. However, the statement provided says that “All required files will be provided from our database on request after acceptance.” This does not seem compatible with the journal’s own requirement, which explicitly states that “data available on request” is not sufficient. This is a serious issue that should be corrected.

There are many awkward or non-idiomatic expressions throughout the paper, such as “investigate into,” “more used,” “in both sides,” “linguistical function,” and “enlighten the language users.” Phrases such as “enlighten the language users” and “further promoting mutual understanding in communication and the language use in general” are too broad and promotional for an academic abstract. The contribution of the study should be stated in more precise scholarly terms. The manuscript would benefit from careful revision by a proficient academic English user.

**********

7. PLOS authors have the option to publish the peer review history of their article (what does this mean?). If published, this will include your full peer review and any attached files.

If you choose “no”, your identity will remain anonymous but your review may still be made public.

**Do you want your identity to be public for this peer review?**  For information about this choice, including consent withdrawal, please see our Privacy Policy.

Reviewer #1: No

**********

[NOTE: If reviewer comments were submitted as an attachment file, they will be attached to this email and accessible via the submission site. Please log into your account, locate the manuscript record, and check for the action link "View Attachments". If this link does not appear, there are no attachment files.]

To ensure your figures meet our technical requirements, please review our figure guidelines: https://journals.plos.org/plosone/s/figures

You may also use PLOS’s free figure tool, NAAS, to help you prepare publication quality figures: https://journals.plos.org/plosone/s/figures#loc-tools-for-figure-preparation.

NAAS will assess whether your figures meet our technical requirements by comparing each figure against our figure specifications.

# Author comment: Author response to Decision Letter 2

**DOI:** 10.1371/journal.pone.0348273.r004

12 Apr 2026

Thank you for all you have done.

Attachment Submitted filename: Response to Reviewer.docx

# Peer review: Decision Letter 2

**DOI:** 10.1371/journal.pone.0348273.r005

**Authors:** Jie Wang

14 Apr 2026

Towards a Behavioral Profile of Degree Adverbs in Mainland and Taiwan Mandarin News Language

PONE-D-26-00164R2

Dear Dr. Zhao,

We’re pleased to inform you that your manuscript has been judged scientifically suitable for publication and will be formally accepted for publication once it meets all outstanding technical requirements.

Within one week, you’ll receive an e-mail detailing the required amendments. When these have been addressed, you’ll receive a formal acceptance letter and your manuscript will be scheduled for publication.

An invoice will be generated when your article is formally accepted. Please note, if your institution has a publishing partnership with PLOS and your article meets the relevant criteria, all or part of your publication costs will be covered. Please make sure your user information is up-to-date by logging into Editorial Manager at Editorial Manager® and clicking the ‘Update My Information' link at the top of the page. For questions related to billing, please contact billing support.

If your institution or institutions have a press office, please notify them about your upcoming paper to help maximize its impact. If they’ll be preparing press materials, please inform our press team as soon as possible -- no later than 48 hours after receiving the formal acceptance. Your manuscript will remain under strict press embargo until 2 pm Eastern Time on the date of publication. For more information, please contact onepress@plos.org.

Kind regards,

Jie Wang, Ph.D.

Academic Editor

PLOS One

Additional Editor Comments (optional):

Reviewers' comments:

Reviewer's Responses to Questions

**Comments to the Author**

1. If the authors have adequately addressed your comments raised in a previous round of review and you feel that this manuscript is now acceptable for publication, you may indicate that here to bypass the “Comments to the Author” section, enter your conflict of interest statement in the “Confidential to Editor” section, and submit your "Accept" recommendation.

Reviewer #1: (No Response)

**********

2. Is the manuscript technically sound, and do the data support the conclusions?

The manuscript must describe a technically sound piece of scientific research with data that supports the conclusions. Experiments must have been conducted rigorously, with appropriate controls, replication, and sample sizes. The conclusions must be drawn appropriately based on the data presented.

Reviewer #1: Yes

**********

3. Has the statistical analysis been performed appropriately and rigorously?

Reviewer #1: Yes

**********

4. Have the authors made all data underlying the findings in their manuscript fully available?

The PLOS Data policy requires authors to make all data underlying the findings described in their manuscript fully available without restriction, with rare exception (please refer to the Data Availability Statement in the manuscript PDF file). The data should be provided as part of the manuscript or its supporting information, or deposited to a public repository. For example, in addition to summary statistics, the data points behind means, medians and variance measures should be available. If there are restrictions on publicly sharing data—e.g. participant privacy or use of data from a third party—those must be specified.

Reviewer #1: Yes

**********

5. Is the manuscript presented in an intelligible fashion and written in standard English?

PLOS ONE does not copyedit accepted manuscripts, so the language in submitted articles must be clear, correct, and unambiguous. Any typographical or grammatical errors should be corrected at revision, so please note any specific errors here.

Reviewer #1: Yes

**********

6. Review Comments to the Author

Please use the space provided to explain your answers to the questions above. You may also include additional comments for the author, including concerns about dual publication, research ethics, or publication ethics. (Please upload your review as an attachment if it exceeds 20,000 characters)

Reviewer #1: After two rounds of review, I am satisfied that the authors have adequately addressed all of my comments and concerns through rigorous revisions and detailed explanations. The revised manuscript demonstrates significant improvement in methodological rigor, theoretical coherence, and overall readability. I have no further major concerns and recommend its acceptance for publication.

**********

7. PLOS authors have the option to publish the peer review history of their article (what does this mean?). If published, this will include your full peer review and any attached files.

If you choose “no”, your identity will remain anonymous but your review may still be made public.

**Do you want your identity to be public for this peer review?**  For information about this choice, including consent withdrawal, please see our Privacy Policy.

Reviewer #1: No

**********

# Editor report: Acceptance letter

**DOI:** 10.1371/journal.pone.0348273.r006

**Authors:** Jie Wang

PONE-D-26-00164R2

PLOS One

Dear Dr. Zhao,

I'm pleased to inform you that your manuscript has been deemed suitable for publication in PLOS One. Congratulations! Your manuscript is now being handed over to our production team.

At this stage, our production department will prepare your paper for publication. This includes ensuring the following:

* All references, tables, and figures are properly cited

* All relevant supporting information is included in the manuscript submission,

* There are no issues that prevent the paper from being properly typeset

You will receive further instructions from the production team, including instructions on how to review your proof when it is ready. Please keep in mind that we are working through a large volume of accepted articles, so please give us a few days to review your paper and let you know the next and final steps.

Lastly, if your institution or institutions have a press office, please let them know about your upcoming paper now to help maximize its impact. If they'll be preparing press materials, please inform our press team within the next 48 hours. Your manuscript will remain under strict press embargo until 2 pm Eastern Time on the date of publication. For more information, please contact onepress@plos.org.

You will receive an invoice from PLOS for your publication fee after your manuscript has reached the completed accept phase. If you receive an email requesting payment before acceptance or for any other service, this may be a phishing scheme. Learn how to identify phishing emails and protect your accounts at https://explore.plos.org/phishing.

If we can help with anything else, please email us at customercare@plos.org.

Thank you for submitting your work to PLOS ONE and supporting open access.

Kind regards,

PLOS ONE Editorial Office Staff

on behalf of

Dr. Jie Wang

Academic Editor

PLOS One
